# Supplementary material for: Stochastic Effects in Autoimmune Dynamics
Source: Front Physiol. 2018 Feb 2;9:45. doi: 10.3389/fphys.2018.00045 (PMC5801658; doi:10.3389/fphys.2018.00045)
Supplement: Supplementary file 1 [file DataSheet1.PDF]

Supplementary Material:
Stochastic effects in autoimmune dynamics

1 SUPPLEMENTARY DATA

Derivation of the van Kampen's system size expansion

As described in Section 2.2 of the main text, the CTMC model based on the transitions probabilities yields the following master equation:

$$\begin{aligned}\frac{dP(\mathbf{n},t)}{dt} =& \{(\varepsilon_1^- - 1)q_1 + (\varepsilon_1^+ - 1)q_2 + (\varepsilon_1^+\varepsilon_2^- - 1)q_3 + (\varepsilon_3^- - 1)q_4 + (\varepsilon_3^+ - 1)q_5 \\ &+ (\varepsilon_3^+\varepsilon_4^- - 1)q_6 + (\varepsilon_3^+\varepsilon_5^- - 1)q_7 + (\varepsilon_3^+\varepsilon_6^- - 1)q_8 + (\varepsilon_2^+ - 1)q_9 + (\varepsilon_4^- - 1)q_{10} \\ &+ (\varepsilon_4^+ - 1)q_{11} + (\varepsilon_5^- - 1)q_{12} + (\varepsilon_5^+ - 1)q_{13} + (\varepsilon_6^- - 1)q_{14} + (\varepsilon_6^+ - 1)q_{15} \\ &+ (\varepsilon_7^- - 1)q_{16} + (\varepsilon_7^+ - 1)q_{17}\}P(\mathbf{n},t), \end{aligned} \tag{S1}$$

where $\mathbf{n} = (n_1, n_2, n_3, n_4, n_5, n_6, n_7)$ is the current state of the system, the coefficients q_i are given by

$$\begin{aligned} &q_1 = b_1n_1 + b_2n_1^2, \quad q_2 = d_1n_1 + d_2n_1^2 + \mu_a n_6 n_1, \quad q_3 = \beta n_1 n_2, \quad q_4 = \lambda_{in}, \\ &q_5 = d_{in}n_3, \quad q_6 = p_1\alpha n_3 n_2, \quad q_7 = p_2\alpha n_3 n_2, \quad q_8 = (1 - p_1 - p_2)\alpha n_3 n_2, \\ &q_9 = (d_F + \mu_F n_5 + \mu_a n_6)n_2, \quad q_{10} = \lambda_r + \rho_1 n_7 n_4, \quad q_{11} = d_r n_4, \quad q_{12} = \rho_2 n_7 n_5, \\ &q_{13} = d_n n_5, \quad q_{14} = \rho_3 n_7 n_6, \quad q_{15} = (d_a + \delta n_4)n_6, \quad q_{16} = \sigma_1 n_5 + \sigma_2 n_6, \quad q_{17} = d_i n_7, \end{aligned}$$

and the operators $\varepsilon_i^\pm$ are defined as follows,

$$\varepsilon_i^\pm f(n_1, n_2, n_3, n_4, n_5, n_6, n_7, t) = f(n_1, ..., n_i \pm 1, ..., n_7, t), \qquad 1 \le i \le 7.$$

If $n_i < 0$ for any $1 \le i \le 7$, then $P(\mathbf{n}, t) = 0$.

To derive the van Kampen's system size expansion of the master equation (S1), we rewrite each $n_i(t)$ in the form

$$n_i(t) = \Omega x_i(t) + \Omega^{1/2}\zeta_i(t), \qquad 1 \le i \le 7,$$

where $\Omega x_i(t) = \mathbb{E}[n_i(t)]$, so $\zeta_i(t)$ represent the fluctuations. Replacing the probability density $P(\mathbf{n}, t)$ by the equivalent probability density $\Pi(\boldsymbol{\zeta}, t)$, i.e. with $\Pi(\boldsymbol{\zeta}, t) = P(\mathbf{n}, t) = P\left(\Omega\mathbf{x} + \Omega^{1/2}\boldsymbol{\zeta}, t\right)$, the left-hand side of the master equation (S1) transforms into

$$\frac{dP(\mathbf{n},t)}{dt} = \frac{\partial \Pi}{\partial t} - \sum_{i=1}^{7} \Omega^{1/2} \frac{dx_i}{dt}\frac{\partial \Pi}{\partial \zeta_i}. \tag{S2}$$

The operators $\varepsilon_i^{\pm}$ and their product now satisfy the following expansions

$$\varepsilon_i^{\pm} = 1 \pm \Omega^{-1/2}\frac{\partial}{\partial\zeta_i} + \frac{1}{2}\Omega^{-1}\frac{\partial^2}{\partial\zeta_i^2} \pm \cdots ,$$

$$\begin{aligned}\varepsilon_i^{+}\varepsilon_j^{-} &= \left(1 + \Omega^{-1/2}\frac{\partial}{\partial\zeta_i} + \frac{1}{2}\Omega^{-1}\frac{\partial^2}{\partial\zeta_i^2} + \cdots\right)\left(1 - \Omega^{-1/2}\frac{\partial}{\partial\zeta_j} + \frac{1}{2}\Omega^{-1}\frac{\partial^2}{\partial\zeta_j^2} - \cdots\right) \\ &= 1 + \Omega^{-1/2}\left(\frac{\partial}{\partial\zeta_i} - \frac{\partial}{\partial\zeta_j}\right) + \Omega^{-1}\left(\frac{1}{2}\frac{\partial^2}{\partial\zeta_i^2} - \frac{\partial^2}{\partial\zeta_i\partial\zeta_j} + \frac{1}{2}\frac{\partial^2}{\partial\zeta_j^2}\right) + \cdots, \quad 1 \le i,j \le 7.\end{aligned} \tag{S3}$$

One can easily obtain/introduce Ω-expansions for all parameters q_i:

$$\begin{aligned}q_1 &= b_1 n_1 + b_2 n_1^2 = b_1\left(\Omega x_1 + \Omega^{1/2}\zeta_1\right) + b_2\left(\Omega^2 x_1^2 + \Omega\zeta_1^2 + 2\Omega^{3/2}x_1\zeta_1\right) \\ &= b_1\left(\Omega x_1 + \Omega^{1/2}\zeta_1\right) + \underbrace{b_2\Omega}_{\widetilde{b}_2}\left(\Omega x_1^2 + \zeta_1^2 + 2\Omega^{1/2}x_1\zeta_1\right) \\ &= \widetilde{b}_2\zeta_1^2 + \left(b_1\zeta_1 + 2\widetilde{b}_2 x_1\zeta_1\right)\Omega^{1/2} + \left(b_1 x_1 + \widetilde{b}_2 x_1^2\right)\Omega,\end{aligned}$$

$$\begin{aligned}q_2 &= d_1 n_1 + d_2 n_1^2 + \mu_a n_6 n_1 \\ &= d_1\left(\Omega x_1 + \Omega^{1/2}\zeta_1\right) + d_2\left(\Omega^2 x_1^2 + \Omega\zeta_1^2 + 2\Omega^{3/2}x_1\zeta_1\right) + \mu_a\left(\Omega x_1 + \Omega^{1/2}\zeta_1\right)\left(\Omega x_6 + \Omega^{1/2}\zeta_6\right) \\ &= d_1\left(\Omega x_1 + \Omega^{1/2}\zeta_1\right) + \underbrace{d_2\Omega}_{\widetilde{d}_2}\left(\Omega x_1^2 + \zeta_1^2 + 2\Omega^{1/2}x_1\zeta_1\right) + \underbrace{\mu_a\Omega}_{\widetilde{\mu}_a}\left(\Omega^{1/2}x_1 + \zeta_1\right)\left(\Omega^{1/2}x_6 + \zeta_6\right) \\ &= d_1\left(\Omega x_1 + \Omega^{1/2}\zeta_1\right) + \widetilde{d}_2\left(\Omega x_1^2 + \zeta_1^2 + 2\Omega^{1/2}x_1\zeta_1\right) + \widetilde{\mu}_a\left(\Omega x_1 x_6 + \Omega^{1/2}x_1\zeta_6 + \Omega^{1/2}x_6\zeta_1 + \zeta_1\zeta_6\right) \\ &= \widetilde{\mu}_a\zeta_1\zeta_6 + \left(d_1\zeta_1 + 2\widetilde{d}_2 x_1\zeta_1 + \widetilde{\mu}_a x_1\zeta_6 + \widetilde{\mu}_a x_6\zeta_1\right)\Omega^{1/2} + \left(d_1 x_1 + \widetilde{d}_2 x_1^2 + \widetilde{\mu}_a x_1 x_6\right)\Omega,\end{aligned}$$

$$\begin{aligned}q_3 &= \beta n_1 n_2 = \beta\left(\Omega x_1 + \Omega^{1/2}\zeta_1\right)\left(\Omega x_2 + \Omega^{1/2}\zeta_2\right) = \underbrace{\beta\Omega}_{\widetilde{\beta}}\left(\Omega^{1/2}x_1 + \zeta_1\right)\left(\Omega^{1/2}x_2 + \zeta_2\right) \\ &= \widetilde{\beta}\left(\Omega x_1 x_2 + \Omega^{1/2}x_1\zeta_2 + \Omega^{1/2}x_2\zeta_1 + \zeta_1\zeta_2\right) = \widetilde{\beta}\zeta_1\zeta_2 + \left(\widetilde{\beta}x_1\zeta_2 + \widetilde{\beta}x_2\zeta_1\right)\Omega^{1/2} + \widetilde{\beta}x_1x_2\Omega,\end{aligned}$$

$$q_4 = \lambda_{in} = \underbrace{\frac{\lambda_{in}}{\Omega}}_{\widetilde{\lambda}_{in}}\Omega = \widetilde{\lambda}_{in}\Omega,$$

$$q_5 = d_{in}n_3 = d_{in}\left(\Omega x_3 + \Omega^{1/2}\zeta_3\right) = d_{in}\zeta_3\Omega^{1/2} + d_{in}x_3\Omega,$$

In a similar way we can easily show

$$q_6 = p_1\widetilde{\alpha}\zeta_2\zeta_3 + \left(p_1\widetilde{\alpha}x_2\zeta_3 + p_1\widetilde{\alpha}x_3\zeta_2\right)\Omega^{1/2} + p_1\widetilde{\alpha}x_2x_3\Omega,$$

$$q_7 = p_2\widetilde{\alpha}\zeta_2\zeta_3 + \left(p_2\widetilde{\alpha}x_2\zeta_3 + p_2\widetilde{\alpha}x_3\zeta_2\right)\Omega^{1/2} + p_2\widetilde{\alpha}x_2x_3\Omega,$$

$$q_8 = (1 - p_1 - p_2)\left[\widetilde{\alpha}\zeta_2\zeta_3 + \widetilde{\alpha}x_2\zeta_3 + \widetilde{\alpha}x_3\zeta_2\Omega^{1/2} + \widetilde{\alpha}x_2x_3\Omega\right],$$

$$\begin{aligned} q_9 =& \left(\widetilde{\mu}_F\zeta_2\zeta_5 + \widetilde{\mu}_a\zeta_2\zeta_6\right) + \left(d_F + \widetilde{\mu}_F x_2\zeta_5 + \widetilde{\mu}_F x_5\zeta_2 + \widetilde{\mu}_a x_2\zeta_6 + \widetilde{\mu}_a x_6\zeta_2\right)\Omega^{1/2} \\ &+ \left(d_F x_2 + \widetilde{\mu}_F x_2x_5 + \widetilde{\mu}_a x_2x_6\right)\Omega, \end{aligned}$$

$$q_{10} = \widetilde{\rho}_1\zeta_4\zeta_7 + \left(\widetilde{\rho}_1x_4\zeta_7 + \widetilde{\rho}_1x_7\zeta_4\right)\Omega^{1/2} + \left(\widetilde{\lambda}_r + \widetilde{\rho}_1x_4x_7\right)\Omega,$$

$$q_{11} = d_r\zeta_4\Omega^{1/2} + d_rx_4\Omega, \quad q_{12} = \widetilde{\rho}_2\zeta_5\zeta_7 + \left(\widetilde{\rho}_2x_5\zeta_7 + \widetilde{\rho}_2x_7\zeta_5\right)\Omega^{1/2} + \widetilde{\rho}_2x_5x_7\Omega,$$

$$q_{13} = d_n\zeta_5\Omega^{1/2} + d_nx_5\Omega, \quad q_{14} = \widetilde{\rho}_3\zeta_6\zeta_7 + \left(\widetilde{\rho}_3x_6\zeta_7 + \widetilde{\rho}_3x_7\zeta_6\right)\Omega^{1/2} + \widetilde{\rho}_3x_6x_7\Omega,$$

$$q_{15} = \widetilde{\delta}\zeta_4\zeta_6 + \left(d_a\zeta_6 + \widetilde{\delta}x_4\zeta_6 + \widetilde{\delta}x_6\zeta_4\right)\Omega^{1/2} + \left(d_ax_6 + \widetilde{\delta}x_4x_6\right)\Omega,$$

$$q_{16} = \left(\sigma_1\zeta_5 + \sigma_2\zeta_6\right)\Omega^{1/2} + (\sigma_1x_5 + \sigma_2x_6)\Omega, \quad q_{17} = d_i\zeta_7\Omega^{1/2} + d_ix_5\Omega,$$

where

$$\lambda_r = \widetilde{\lambda}_r\Omega, \quad \mu_F = \frac{\widetilde{\mu}_F}{\Omega}, \quad \alpha = \frac{\widetilde{\alpha}}{\Omega}, \quad \delta = \frac{\widetilde{\delta}}{\Omega}, \quad \rho_i = \frac{\widetilde{\rho}_i}{\Omega},\ i = 1, 2, 3.$$

Substituting expressions (S2), (S3) and q_i's into the master equation (S1) shows that the left-hand side of the equation only contains terms of the order $\Omega^{1/2}$ and Ω^0, while the right-hand side has terms of the order $\Omega^{1/2}$, Ω^0, and $\Omega^{-n/2}$, for $n \in \mathbb{N}$. To derive a linear Fokker-Planck equation, we ignore the terms of order $\Omega^{-n/2}$, for $n \in \mathbb{N}$.

Considering the terms of order $\Omega^{1/2}$, i.e. only the terms that are proportional to $\partial\Pi/\partial\zeta_i$, yields

$$
\begin{aligned}
-\Omega^{1/2}\frac{dx_1}{dt}\frac{\partial\Pi}{\partial\zeta_1} &= \left(-\Omega^{-1/2}\frac{\partial}{\partial\zeta_1}\right)\left[\left(b_1x_1+\widetilde{b}_2x_1^2\right)\Omega\right]\Pi+\left(\Omega^{-1/2}\frac{\partial}{\partial\zeta_1}\right)\left[\left(d_1x_1+\widetilde{d}_2x_1^2+\widetilde{\mu}_ax_1x_6\right)\Omega\right]\Pi\\
&+\left(\Omega^{-1/2}\frac{\partial}{\partial\zeta_1}\right)\left[\widetilde{\beta}x_1x_2\Omega\right]\Pi,\\
-\Omega^{1/2}\frac{dx_2}{dt}\frac{\partial\Pi}{\partial\zeta_2} &= \left(-\Omega^{-1/2}\frac{\partial}{\partial\zeta_2}\right)\left[\widetilde{\beta}x_1x_2\Omega\right]\Pi+\left(\Omega^{-1/2}\frac{\partial}{\partial\zeta_2}\right)\left[\left(d_Fx_2+\widetilde{\mu}_Fx_2x_5+\widetilde{\mu}_ax_2x_6\right)\Omega\right]\Pi,\\
-\Omega^{1/2}\frac{dx_3}{dt}\frac{\partial\Pi}{\partial\zeta_3} &= \left(-\Omega^{-1/2}\frac{\partial}{\partial\zeta_3}\right)\left[\widetilde{\lambda}_{in}\Omega\right]\Pi+\left(\Omega^{-1/2}\frac{\partial}{\partial\zeta_3}\right)\left[d_{in}x_3\Omega\right]\Pi+\left(\Omega^{-1/2}\frac{\partial}{\partial\zeta_3}\right)\left[p_1\widetilde{\alpha}x_2x_3\Omega\right]\Pi\\
&\left(\Omega^{-1/2}\frac{\partial}{\partial\zeta_3}\right)\left[p_2\widetilde{\alpha}x_2x_3\Omega\right]\Pi+\left(\Omega^{-1/2}\frac{\partial}{\partial\zeta_3}\right)\left[(1-p_1-p_2)\widetilde{\alpha}x_2x_3\Omega\right]\Pi,\\
-\Omega^{1/2}\frac{dx_4}{dt}\frac{\partial\Pi}{\partial\zeta_4} &= \left(-\Omega^{-1/2}\frac{\partial}{\partial\zeta_4}\right)\left[p_1\widetilde{\alpha}x_2x_3\Omega\right]\Pi+\left(-\Omega^{-1/2}\frac{\partial}{\partial\zeta_4}\right)\left[\left(\widetilde{\lambda}_r+\widetilde{\rho}_1x_4x_7\right)\Omega\right]\Pi\\
&+\left(\Omega^{-1/2}\frac{\partial}{\partial\zeta_4}\right)\left[d_rx_4\Omega\right]\Pi,\\
-\Omega^{1/2}\frac{dx_5}{dt}\frac{\partial\Pi}{\partial\zeta_5} &= \left(-\Omega^{-1/2}\frac{\partial}{\partial\zeta_5}\right)\left[p_2\widetilde{\alpha}x_2x_3\Omega\right]\Pi+\left(-\Omega^{-1/2}\frac{\partial}{\partial\zeta_5}\right)\left[\widetilde{\rho}_2x_5x_7\Omega\right]\Pi\\
&-\left(\Omega^{-1/2}\frac{\partial}{\partial\zeta_5}\right)\left[d_nx_5\Omega\right]\Pi,\\
-\Omega^{1/2}\frac{dx_6}{dt}\frac{\partial\Pi}{\partial\zeta_6} &= \left(-\Omega^{-1/2}\frac{\partial}{\partial\zeta_6}\right)\left[(1-p_1-p_2)\widetilde{\alpha}x_2x_3\Omega\right]\Pi+\left(-\Omega^{-1/2}\frac{\partial}{\partial\zeta_6}\right)\left[\widetilde{\rho}_3x_6x_7\Omega\right]\Pi\\
&+\left(\Omega^{-1/2}\frac{\partial}{\partial\zeta_6}\right)\left[\left(d_ax_6+\widetilde{\delta}x_4x_6\right)\Omega\right]\Pi,\\
-\Omega^{1/2}\frac{dx_7}{dt}\frac{\partial\Pi}{\partial\zeta_7} &= \left(-\Omega^{-1/2}\frac{\partial}{\partial\zeta_7}\right)\left[(\sigma_1x_5+\sigma_2x_6)\Omega\right]\Pi+\left(\Omega^{-1/2}\frac{\partial}{\partial\zeta_7}\right)\left[d_ix_5\Omega\right]\Pi.
\end{aligned}
$$

After simplification, we obtain the following system of equations that describes macroscopic behaviour of the model

$$
\begin{aligned}
\frac{dx_1}{dt} &= b_1x_1 + \widetilde{b}_2x_1^2 - d_1x_1 - \widetilde{d}_2x_1^2 - \widetilde{\beta}x_1x_2 - \widetilde{\mu}_ax_1x_6,\\
\frac{dx_2}{dt} &= \widetilde{\beta}x_1x_2 - d_Fx_2 - \widetilde{\mu}_Fx_2x_5 - \widetilde{\mu}_ax_2x_6,\\
\frac{dx_3}{dt} &= \widetilde{\lambda}_{in} - d_{in}x_3 - \widetilde{\alpha}x_2x_3,\\
\frac{dx_4}{dt} &= \widetilde{\lambda}_r - d_rx_4 + p_1\widetilde{\alpha}x_2x_3 + \widetilde{\rho}_1x_4x_7,\\
\frac{dx_5}{dt} &= p_2\widetilde{\alpha}x_2x_3 - d_nx_5 + \widetilde{\rho}_2x_5x_7,\\
\frac{dx_6}{dt} &= (1-p_1-p_2)\widetilde{\alpha}x_2x_3 - d_ax_6 - \widetilde{\delta}x_4x_6 + \widetilde{\rho}_3x_6x_7,\\
\frac{dx_7}{dt} &= \sigma_1x_5 + \sigma_2x_6 - d_ix_7.
\end{aligned}
\tag{S4}
$$

Terms of order Ω^0 give the following Fokker-Planck equation

$$
\begin{aligned}
\frac{\partial \Pi}{\partial t} = -\Bigg[& \left(b_1 + 2\widetilde{b}_2x_1 - d_1 - 2\widetilde{d}_2x_1 - \widetilde{\mu}_ax_6 - \widetilde{\beta}x_2\right)\frac{\partial(\zeta_1\Pi)}{\partial\zeta_1} - \widetilde{\beta}x_1\frac{\partial(\zeta_2\Pi)}{\partial\zeta_1} - \widetilde{\mu}_ax_1\frac{\partial(\zeta_6\Pi)}{\partial\zeta_1}\\
& + \widetilde{\beta}x_2\frac{\partial(\zeta_1\Pi)}{\partial\zeta_2} + \left(\widetilde{\beta}x_1 - d_F - \widetilde{\mu}_Fx_5 - \widetilde{\mu}_ax_6\right)\frac{\partial(\zeta_2\Pi)}{\partial\zeta_2} - \widetilde{\mu}_Fx_2\frac{\partial(\zeta_5\Pi)}{\partial\zeta_2} - \widetilde{\mu}_ax_2\frac{\partial(\zeta_6\Pi)}{\partial\zeta_2}\\
& - \widetilde{\alpha}x_3\frac{\partial(\zeta_2\Pi)}{\partial\zeta_3} - (d_{in} + \widetilde{\alpha}x_2)\frac{\partial(\zeta_3\Pi)}{\partial\zeta_3} + p_1\widetilde{\alpha}x_3\frac{\partial(\zeta_2\Pi)}{\partial\zeta_4} + p_1\widetilde{\alpha}x_2\frac{\partial(\zeta_3\Pi)}{\partial\zeta_4} + (\widetilde{\rho}_1x_7 - d_r)\frac{\partial(\zeta_4\Pi)}{\partial\zeta_4}\\
& + \widetilde{\rho}_1x_4\frac{\partial(\zeta_7\Pi)}{\partial\zeta_4} + p_2\widetilde{\alpha}x_3\frac{\partial(\zeta_2\Pi)}{\partial\zeta_5} + p_2\widetilde{\alpha}x_2\frac{\partial(\zeta_3\Pi)}{\partial\zeta_5} + (\widetilde{\rho}_2x_7 - d_n)\frac{\partial(\zeta_5\Pi)}{\partial\zeta_5} + \widetilde{\rho}_2x_5\frac{\partial(\zeta_7\Pi)}{\partial\zeta_5}\\
& + (1-p_1-p_2)\widetilde{\alpha}x_3\frac{\partial(\zeta_2\Pi)}{\partial\zeta_6} + (1-p_1-p_2)\widetilde{\alpha}x_2\frac{\partial(\zeta_3\Pi)}{\partial\zeta_6} - \widetilde{\delta}x_6\frac{\partial(\zeta_4\Pi)}{\partial\zeta_6}\\
& + \left(\widetilde{\rho}_3x_7 - d_a - \widetilde{\delta}\right)x_4\frac{\partial(\zeta_6\Pi)}{\partial\zeta_6} + \widetilde{\rho}_3x_6\frac{\partial(\zeta_7\Pi)}{\partial\zeta_6} + \sigma_1\frac{\partial(\zeta_5\Pi)}{\partial\zeta_7} + \sigma_2\frac{\partial(\zeta_6\Pi)}{\partial\zeta_7} - d_i\frac{\partial(\zeta_7\Pi)}{\partial\zeta_7}\Bigg]\\
+\frac{1}{2}\Bigg\{ & \left(b_1x_1 + \widetilde{b}_2x_1^2 + d_1x_1 + \widetilde{d}_2x_1^2 + \widetilde{\beta}x_1x_2 + \widetilde{\mu}_ax_1x_6\right)\frac{\partial^2\Pi}{\partial\zeta_1^2} - 2\widetilde{\beta}x_1x_2\frac{\partial^2\Pi}{\partial\zeta_1\partial\zeta_2}\\
& + \left(\widetilde{\beta}x_1x_2 + d_Fx_2 + \widetilde{\mu}_Fx_2x_5 + \widetilde{\mu}_ax_2x_6\right)\frac{\partial^2\Pi}{\partial\zeta_2^2} + \left(\widetilde{\lambda}_{in} + d_{in}x_3 + \widetilde{\alpha}x_2x_3\right)\frac{\partial^2\Pi}{\partial\zeta_3^2}\\
& - 2p_1\widetilde{\alpha}x_2x_3\frac{\partial^2\Pi}{\partial\zeta_3\partial\zeta_4} - 2p_2\widetilde{\alpha}x_2x_3\frac{\partial^2\Pi}{\partial\zeta_3\partial\zeta_5} - 2(1-p_1-p_2)\widetilde{\alpha}x_2x_3\frac{\partial^2\Pi}{\partial\zeta_3\partial\zeta_6}\\
& + \left(\widetilde{\lambda}_r + d_rx_4 + p_1\widetilde{\alpha}x_2x_3 + \widetilde{\rho}_1x_4x_7\right)\frac{\partial^2\Pi}{\partial\zeta_4^2} + \left(p_2\widetilde{\alpha}x_2x_3 + d_nx_5 + \widetilde{\rho}_2x_5x_7\right)\frac{\partial^2\Pi}{\partial\zeta_5^2}\\
& + \left[(1-p_1-p_2)\widetilde{\alpha}x_2x_3 + d_ax_6 + \widetilde{\delta}x_4x_6 + \widetilde{\rho}_3x_6x_7\right]\frac{\partial^2\Pi}{\partial\zeta_6^2} + (\sigma_1x_5 + \sigma_2x_6 + d_ix_7)\frac{\partial^2\Pi}{\partial\zeta_7^2}\Bigg\}.
\end{aligned}
$$

This equation can be equivalently rewritten in the form

$$\frac{\partial \Pi(\boldsymbol{\zeta}, t)}{\partial t} = -\sum_{i,j} A_{ij} \frac{\partial}{\partial \zeta_i}(\zeta_j \Pi) + \frac{1}{2} \sum_{i,j} B_{ij} \frac{\partial^2 \Pi}{\partial \zeta_i \partial \zeta_j},$$

where A is the Jacobian matrix of system (S4)

$$A = \begin{pmatrix} b_1 + 2\widetilde{b}_2 x_1 - d_1 - 2\widetilde{d}_2 x_1 - \widetilde{\mu}_a x_6 - \widetilde{\beta} x_2 & -\widetilde{\beta} x_1 & 0 & 0 & 0 & -\widetilde{\mu}_a x_1 & 0 \\ \widetilde{\beta} x_2 & \widetilde{\beta} x_1 - d_F - \widetilde{\mu}_F x_5 - \widetilde{\mu}_a x_6 & 0 & 0 & -\widetilde{\mu}_F x_2 & -\widetilde{\mu}_a x_2 & 0 \\ 0 & -\widetilde{\alpha} x_3 & -d_{in} - \widetilde{\alpha} x_2 & 0 & 0 & 0 & 0 \\ 0 & p_1 \widetilde{\alpha} x_3 & p_1 \widetilde{\alpha} x_2 & \widetilde{\rho}_1 x_7 - d_r & 0 & 0 & \widetilde{\rho}_1 x_4 \\ 0 & p_2 \widetilde{\alpha} x_3 & p_2 \widetilde{\alpha} x_2 & 0 & \widetilde{\rho}_2 x_7 - d_n & 0 & \widetilde{\rho}_2 x_5 \\ 0 & (1 - p_1 - p_2)\widetilde{\alpha} x_3 & (1 - p_1 - p_2)\widetilde{\alpha} x_2 & -\widetilde{\delta} x_6 & 0 & \widetilde{\rho}_3 x_7 - d_a - \widetilde{\delta} x_4 & \widetilde{\rho}_3 x_6 \\ 0 & 0 & 0 & 0 & \sigma_1 & \sigma_2 & -d_i \end{pmatrix}$$

B is a 7×7 symmetric matrix given by

$$B_{ij} = \begin{cases} b_1 x_1 + \widetilde{b}_2 x_1^2 + d_1 x_1 + \widetilde{d}_2 x_1^2 + \widetilde{\beta} x_1 x_2 + \widetilde{\mu}_a x_1 x_6, & \text{if } (i,j) = (1,1), \\ \widetilde{\beta} x_1 x_2 + d_F x_2 + \widetilde{\mu}_F x_2 x_5 + \widetilde{\mu}_a x_2 x_6, & \text{if } (i,j) = (2,2), \\ \widetilde{\lambda}_{in} + d_{in} x_3 + \widetilde{\alpha} x_2 x_3, & \text{if } (i,j) = (3,3), \\ \widetilde{\lambda}_r + d_r x_4 + p_1 \widetilde{\alpha} x_2 x_3 + \widetilde{\rho}_1 x_4 x_7, & \text{if } (i,j) = (4,4), \\ p_2 \widetilde{\alpha} x_2 x_3 + d_n x_5 + \widetilde{\rho}_2 x_5 x_7, & \text{if } (i,j) = (5,5), \\ (1 - p_1 - p_2)\widetilde{\alpha} x_2 x_3 + d_a x_6 + \widetilde{\delta} x_4 x_6 + \widetilde{\rho}_3 x_6 x_7, & \text{if } (i,j) = (6,6), \\ \sigma_1 x_5 + \sigma_2 x_6 + d_i x_7, & \text{if } (i,j) = (7,7), \\ -\widetilde{\beta} x_1 x_2, & \text{if } (i,j) = (1,2) \text{ or } (2,1), \\ -p_1 \widetilde{\alpha} x_2 x_3, & \text{if } (i,j) = (3,4) \text{ or } (4,3), \\ -p_2 \widetilde{\alpha} x_2 x_3, & \text{if } (i,j) = (3,5) \text{ or } (5,3), \\ -(1 - p_1 - p_2)\widetilde{\alpha} x_2 x_3, & \text{if } (i,j) = (3,6) \text{ or } (6,3), \\ 0, & \text{otherwise.} \end{cases}$$
